# Treatment of Disorders of the Teeth during Pregnancy

**Published:** 1892-10

**Authors:** 


### Treatment of Disorders of the Teeth during Pregnancy.

In the Birmingham *Medical Review*, July, 1892, Elliott describes several diseased conditions of the teeth and gums occurring during pregnancy. In anæmic patients the gums become thin, pale, and shrivelled in appearance and retracted from the edge of the teeth; a prominent ridge is often seen near the free border. In other cases the gums are full and reddened, apparently deeply congested and containing pus, which exudes on pressure.

An important factor in producing caries of the teeth is the altered condition of the secretions; their reaction becomes acid instead of alkaline, which favors markedly the disintegration of the teeth. Caries of the teeth is often accompanied by a brownish discoloration on the labial surface of the tooth following the outline of the gum; the enamel is brittle and opaque. The upper bicuspids and molars are often carious, the dentine being softened but not discolored. The lesion in these cases is caused by the fact that the tongue lies frequently in contact with these teeth, and the acid mucus and matter vomited come frequently in contact. In anæmic patients "white" or soft caries accompanied by absorption of the alveolar process is observed; the teeth become so loosened as to frequently come out.

Neuralgic pain in or about the teeth is sometimes so severe as to delay labor. Hysterical pain in the teeth sometimes extends to several, it may be fixed in two sound teeth, or may shift from side to side.

As regards treatment, quinine and opium may be used when the gums are anæmic; ammonium chloride and aconite when full and congested. Chlorate of potassium and potassium bromide are efficacious in irritable gums and teeth. Equal parts of charcoal and prepared chalk are useful as a local application to irritable teeth and gums. In reflex pain in the teeth and gums a blister three by one inch in size applied over the fourth and fifth dorsal vertebræ, often affords relief. When pain in the teeth is constant and depressing, removal under an anæsthetic is indicated.—*Amer. Journ. of Med. Sciences.*

